# Supplementary material for: A Genome-Wide Identification and Expression Pattern of LMCO Gene Family from Turnip (Brassica rapa L.) under Various Abiotic Stresses
Source: Plants (Basel). 2023 May 7;12(9):1904. doi: 10.3390/plants12091904 (PMC10180887; doi:10.3390/plants12091904)
Supplement: Supplementary file 1 [file plants-12-01904-s001.zip › plants-2344335-supplementary.pdf]

Table S1. Groups base on the phylogenetic tree and motifs analysis of Arabidopsis and Brassica LMCO genes

S.No	*Arabidopsis* LMCO genes	*Brassica rapa* LMCO genes	Group
1	At2g29130 (At-Lac2)	XP_009144396.1 (Br-Lac2)	**1**
2	At5g60020 (At-Lac17)	XP_009131958.1 (Br-Lac17)	
3	At2g38080 (At-Lac4)	XP_033148764.1 (Br-Lac4)	**2**
4	At5g01190 (At-Lac10)	XP_009125430.1 (Br-Lac10)	
5	At5g03260 (At-Lac11)	XP_009125488.1 ((Br-Lac11)	
6	At5g58910 (At-Lac16)	XP_009120399.1 (Br-Lac16)	
7		XP_009146739 (Br-Lac22)	
8	At2g30210 (At-Lac3)	XP_009144319.1 (Br-Lac3)	3
9	At2g40370 (At-Lac5)	XP_009143220.1 (Br-Lac5)	
10	At5g05390 (At-Lac12)	XP_009125599.1 (Br-Lac12)	
11	At5g07130 (At-Lac13)	XP_009122370.1 (Br-Lac13)	
12	At5g09360 (At-Lac14)	XP_009122510.1 (Br-Lac14)	4
13	At5g48100 (At-Lac15)	XP_009129702.1 (Br-Lac15)	
14	At3g09220 (At-Lac7)	XP_009146941.1 (Br-Lac7)	5
15	At5g01040 (At-Lac8)	XP_033142360.1 (Br-Lac8)	
16	At5g01050 (At-Lac9)	XP_009132200.3 (Br-Lac9)	
17	At1g18140 (At-Lac1)	XP_009110363.1 (Br-Lac1)	6
18	At2g46570 (At-Lac6)	XP_009142428.1 (Br-Lac6)	7

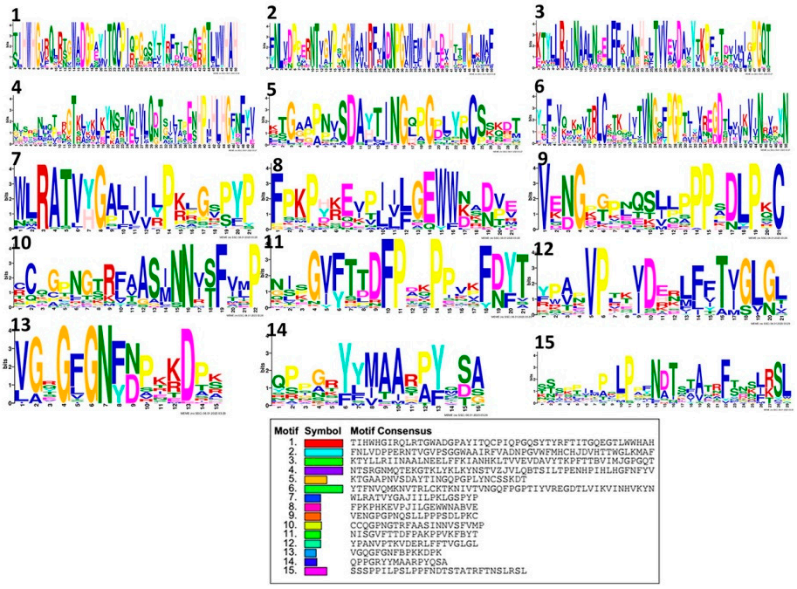

Figure S1. Fifteen conserved motifs were identified in *Arabidopsis* and *Brassica* LMCO proteins using the MEME tool and MEME suite web server to acquire the logos.

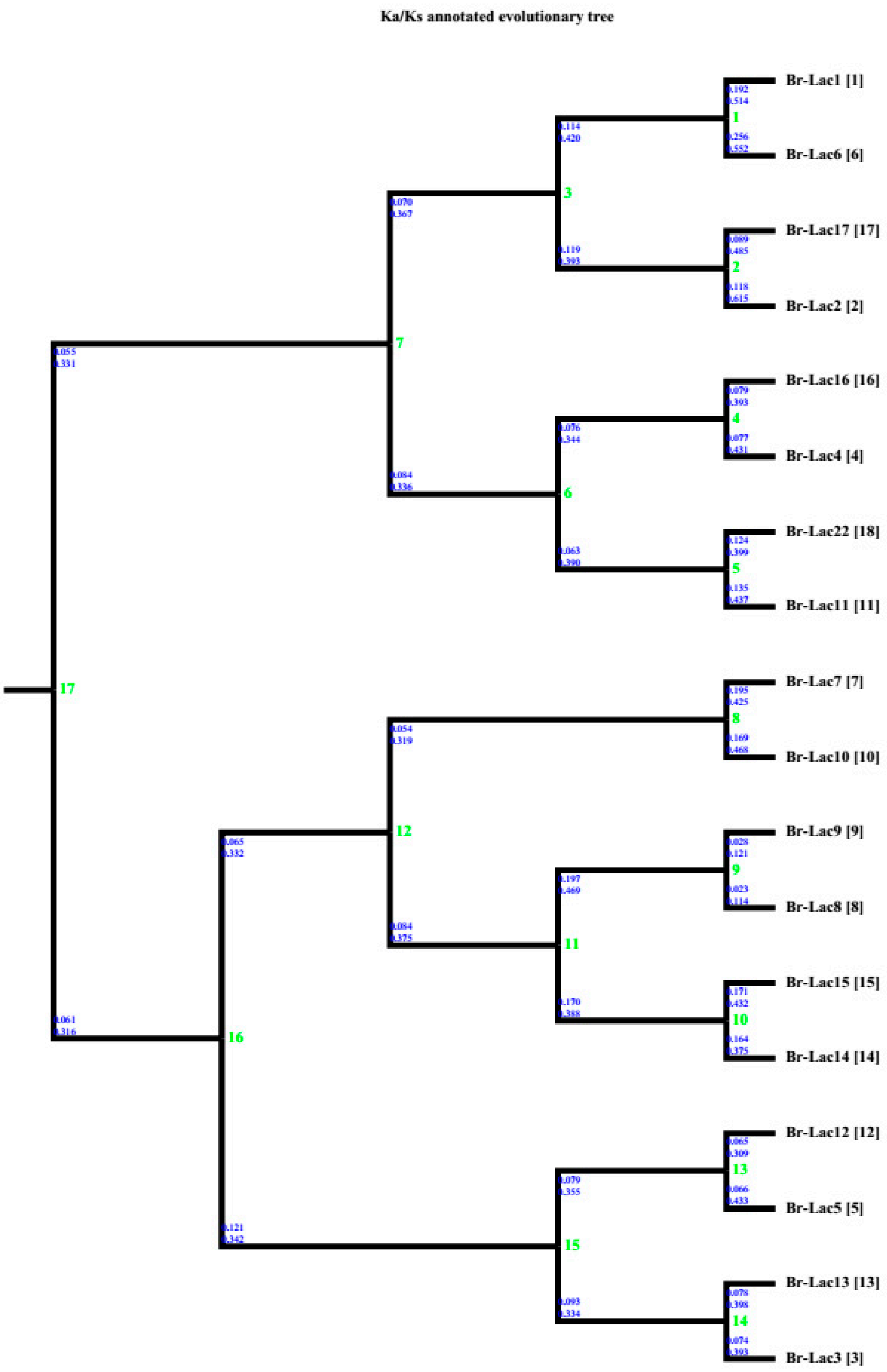

Figure S2. Pair of paralogous Br-Lac genes according to the phylogenetic tree server.

Table S2. List of LMCO genes primers for qRT-PCR.

S.no	Gene name	Sequence ID	
1	Br-Lac-1	F	GAGGATGGGCGGCGATTAGGAT
		R	CACGAGGAGGACGAAGCAGAGT
2	Br-Lac-2	F	AACTTACGGCATCAACGCAGAA
		R	TGTCACATACGACGGTCCATCC
3	Br-Lac-3	F	TCGTCTCGGCTCTCCTTATCCA
		R	GGTGGTTGGCGACTGAGAAGAA
4	Br-Lac-4	F	CAAGCCAACCGTGACCGTGAA
		R	ATGTGAGCGTGCCACCAGAGA
5	Br-Lac-5	F	CACTGGCACGGTGTGAGACAA
		R	CGGAGGAAGGACGAGGAGTGAA
6	Br-Lac-6	F	TGTCTCCTCCTTGCTCCGTCTT
		R	GCACAGCCTGGTGAGTCTCATT
7	Br-Lac-7	F	CCACCAGAGGCGTCATCCACTA
		R	GAGCAGTTGGCGTGTCGAAGAA
8	Br-Lac-8	F	ACGCACTTCTCACGGCTGAC
		R	TATGGTCCACCCACGAGGCTAG
9	Br-Lac-9	F	CGGCACTCAACACTCACCTCT
		R	CGGCGTCAAGATCATCACATCG
10	Br-Lac-10	F	CCTTCACCACCGACACAATCCT
		R	GAATGGAGCGGCGGCGATTA
11	Br-Lac-11	F	GCCTCCGATGTCAGATGCTCAT
		R	CGATCTCCACCACGGTCATGTC
12	Br-Lac-12	F	TGTTCCCTTCTCTCCGCCTCAT
		R	TCTGCCTCACACCATGCCAATG
13	Br-Lac-13	F	CTGACAGTGGTTGCTGCGGATG
		R	AGTAGTGTGCTGGCGGTTGGT
14	Br-Lac-14	F	CGCACAAGCCAAGATTCATCGT
		R	AACGCTCGCCTGGTCTAATCG
15	Br-Lac-15	F	GAGGTTGGTGTCGGGTTTGGT
		R	GGAGGCAAGTTAGGAGGTGGAG
16	Br-Lac-16	F	TCTCGCCAACCACCGTTCACT
		R	TCCATCTGCCCAACCTGTCCTC
17	Br-Lac-17	F	TACGGTCTGGTTGGGCTGATGG
		R	GCGTTGCTTGGCGAATGATTGC
18	Br-Lac-22	F	TAACCTCACCAACAGCGTCAGC
		R	TGCCATAGAAGCGTTCCTCGTT
